# Marital status independently predicts gastric cancer survival after surgical resection--an analysis of the SEER database

**DOI:** 10.18632/oncotarget.7107

**Published:** 2016-01-31

**Authors:** Rong-liang Shi, Qian Chen, Zhen Yang, Gaofeng Pan, Ziping Zhang, WeiHua Wang, Shaoqun Liu, Dongbin Zhang, Daowen Jiang, Weiyan Liu

**Affiliations:** ^1^ Department of General Surgery, Minhang Hospital, Fudan University, Shanghai, People's Republic of China; ^2^ Department of Head and Neck Surgery, Fudan University Shanghai Cancer Center, Shanghai, People's Republic of China; ^3^ Department of Oncology, Shanghai Medical College, Fudan University, Shanghai, People's Republic of China; ^4^ Department of Thoracic Surgery, Minhang Hospital, Fudan University, Shanghai, People's Republic of China

**Keywords:** gastric cancer, marital status, SEER, survival analysis

## Abstract

Marital status was found to be an independent prognostic factor for survival in various cancer types, but it hasn't been studied in gastric cancer. The Surveillance, Epidemiology and End Results database was used to compare survival outcomes with marital status. A total of 16,106 eligible patients were identified. Patients in the widowed group had the highest proportion of women, more common site of stomach, more prevalence of elderly patients, higher percentage of adenocarcinoma, and more tumors at localized stage (*P* < 0.05). Patients in married group had better 5year cause-specific survival (CSS) than those unmarried (*P* < 0.05). Further analysis showed that widowed patients always presented the lowest CSS compared with that of other groups. Widowed patients had 7.1% reduction in 5-year CSS compared with married patients at Localized stage (77.2% *vs* 70.1%, *P* < 0.001), 9.6% reduction at Regional stage (38.2% *vs* 28.6%, *P* < 0.001), and 4.7% reduction at Distant stage (13.3% *vs* 8.6%, *P* < 0.001). These results showed that unmarried patients were at greater risk of cancer specific mortality. Despite favorable clinicpathological characteristics, widowed patients were at highest risk of death compared with other groups.

## INTRODUCTION

Gastric cancer represents a major cause of cancer mortality because of its poor prognosis [1]. The only potentially curative treatment for gastric cancer is complete resection (R0). However, even after surgical management, the 5-year overall survival rate is only about 20% in series from the United States [2]. Several parameters could be used to predict survival outcomes in patients with gastric, including clinicopathological factors, adjuvant therapy, socioeconomic status, and psychiatric supports, such as, marital status. Married persons enjoy overall better health and increased life expectancy compared with the unmarried (divorced, separated, never married) [3-5]. Researches also indicate a survival advantage for married persons living with a chronic disease such as cancer. Accumulated studies have shown that marital status is an independent prognostic factor of survival in various cancer types [6-12]. In a larger population-based study on data from the Surveillance, Epidemiology and End Results (SEER) database indicated that unmarried patients are at significantly higher risk of presentation with metastatic cancer, undertreatment, and death resulting from their cancer in ten leading causes of cancer-related death [6]. To our knowledge, the impact of marital status on gastric cancer survival has not been previously studied. Data does exist to suggest that divorce, widowhood, and living alone increase the risk of each subtype of esophageal and gastric cancer [13], however, the analysis has not been extended to cancer outcome. Given that gastric cancer is one of the most common malignancies with high cancer-related deaths and marriage is an important aspect of adult life, it is important to explore the relationship between marital status and gastric cancer survival outcomes and the potential underlying mechanisms. In this study, we used data from the SEER cancer-registry program of individuals diagnosed between 2004 and 2012 to explore the impact of marital status on gastric cancer cause specific survival (CSS) in patients after surgical resection.

## RESULTS

### Patient baseline characteristics

A total of 16,106 eligible patients were identified during the 9-year study period, including 10,178 male and 5,928 female patients. Of these, 10,273 (63.8%) were married, 2,349 (14.6%) were widowed, and 2,072(12.9%) were single. The 175 (1.1%) individuals who were separated and 1,237(7.7%) who were divorced were grouped together in the divorced/separated group in our study [11]. Patients in the widowed group had the highest proportion of women, more common site of stomach, more prevalence of elderly patients (> 60 years), and more tumors at Localized stage, higher percentage of adenocarcinoma, all of which were statistically significant (*P* < 0.001). Patient demographics and pathological features are summarized in Table 1.

**Table 1:** Baseline demographic and tumor characteristics of patients in SEER database

	Total	Married	Widowed	Single	Divorced/Separated	*P* value
Characteristic	(n=16106)	(n=10273) N(%)	(n=2349) N(%)	(n=2072) N(%)	(n=1412) N(%)	
Sex						<0.001
Male	10178	7465(72.7)	684(29.1)	1253(60.5)	776(55.0)	
Female	5928	2808(27.3)	1665(70.9)	819(39.5)	636(45.0)	
Primary Site						<0.001
Stomach	11638	7087(69.0)	1986(84.5)	1568(75.7)	997(70.6)	
Gastroesophageal Junction	5928	3186(31.0)	363(15.5)	504(24.3)	415(29.4)	
Age						<0.001
≦60	5191	3505(34.1)	106(4.5)	1028(49.6)	552(39.1)	
>60	10915	6768(65.9)	2243(95.5)	1044(50.4)	860(60.9)	
Race						<0.001
White	10869	7090(69.0)	1552(66.1)	1280(61.8)	947(67.1)	
Black	2007	884(8.6)	342(14.6)	510(24.6)	271(19.2)	
Other*	3177	2263(22.0)	448(19.1)	279(13.5)	187(13.2)	
Unknown	53	36(0.4)	7(0.3)	3(0.1)	7(0.5)	
Pathological grading						<0.001
I /II	5179	3234(31.5)	862(36.7)	634(30.6)	449(31.8)	
III/IV	9880	6362(61.9)	1354(57.6)	1290(62.3)	874(61.9)	
Unknown	1047	677(6.6)	133(5.7)	148(7.1)	89(6.3)	
Histotype						<0.001
Adenocarcinoma	12047	7659(74.6)	1883(80.2)	1466(70.8)	1039(73.6)	
Mucinous/Signet ring cell	4059	2614(25.4)	466(19.8)	606(29.2)	373(26.4)	
SEER stage						<0.001
Localized	5621	3530(34.4)	930(39.6)	671(32.4)	490(34.7)	
Regional	7723	4966(48.3)	1062(45.2)	1024(49.4)	671(47.5)	
Distant	2578	1665(16.2)	330(14.0)	347(16.7)	236(16.7)	
Unstaged	184	112 (1.1)	27(1.1)	30(1.4)	15(1.1)	

* Other includes American Indian/Alaska native, Asian/Pacific Islander, etc.

### Effect of marital status on CSS in the SEER database

**Figure 1:**
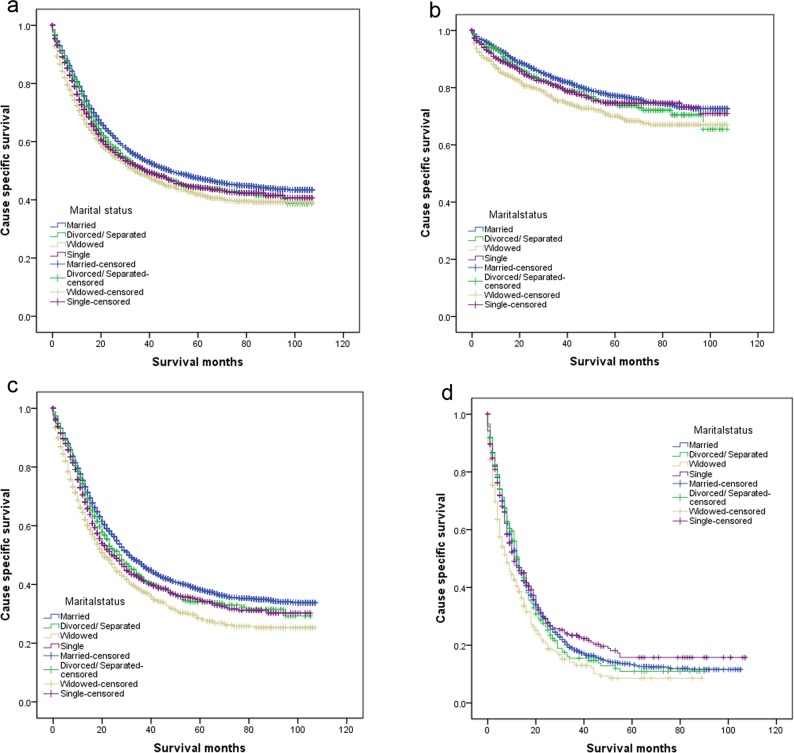
Survival curves in gastric patients according to marital status **a.** All stage; χ^2^ = 49.006, *P* < 0.001; **b.** Localized: χ^2^ = 25.356, *P* < 0.001; **c.** Regional: χ^2^ = 54.197, *P* < 0.001; **d.** Distant: χ^2^ = 20.161, *P* < 0.001.

The overall 5-year CSS was 47.5% in the married group,42.0% in the widowed group,44.4% in the never married group, and 44.4% in the divorced/separated group, which were all significantly different according to the univariate log-rank test (*P* < 0.001) (Table 2, Figure 1a). Additionally, tumor located at gastroesophageal junction (*P* = 0.035), elderly patients (*P* = 0.001), male sex (*P* < 0.001), black ethnicity (*P* < 0.001), poor or undifferentiated tumor grade (*P* < 0.001), mucinous/signet-ring cancer (*P* < 0.001), and advanced SEER stage (*P* < 0.001) were identified as significant risk factors for poor survival on univariate analysis (Table 2).

**Table 2:** Univariate and multivariate survival analysis for evaluating the influence of marital status on gastric cancer cause-specific survival in SEER database

		Univariate analysis		Multivariate analysis	
Variable	5-year CCS	Log rank χ^2^ test	*P*	HR(95%CI)	*P*
**Primary Site**		4.460	0.035		0.881
Stomach	46.5%			Reference	
Gastroesophageal Junction	44.6%			1.004(0.949-1.062)	
**Sex**		1.515	0.218		NI
Male	46.0%				
Female	46.0%				
**Age**		10.218	0.001		<0.001
≦60	46.2%			Reference	
>60	46.9%			1.339(1.269-1.412)	
**Race**		87.890	<0.001		
White	44.1%			Reference	<0.001
Black	43.1%			1.045(0.971-1.123)	
Other*	54.1%			0.785(0.735-0.839)	
**Grade**		518.723	<0.001		<0.001
I / II	59.2%			Reference	
III/ IV	38.2%			1.430(1.349-1.517)	
Unknown	56.9%			1.208 (1.074-1.358)	
**Histotype**		160.754	<0.001		<0.001
Adenocarcinoma	49.2%			Reference	
Mucinous/signet ring cell	36.8%			1.175(1.113-1.241)	
**SEER Stage**		3309.783	<0.001		<0.001
Localized	75.6%			Reference	
Regional	36.1%			3.379 (3.153-3.622)	
Distant	12.8%			7.269(6.725-7.857)	
Unstaged	36.6%			3.473(2.781-4.3361)	
**Marital Status**		49.006	<0.001		<0.001
Married	47.5%			Reference	
Windowed	42.0%			1.127(1.205-1.382)	
Never married	44.4%			1.290(1.205-1.382)	
Divorced/Separated	44.4%			1.082(0.994-1.179)	

* Other includes American Indian/Alaska native, Asian/Pacific Islander, and unknown.

NI: not included in the multivariate survival analysis.

When multivariate analysis with Cox regression was performed, six variables were validated as independent prognostic factors,including age (> 60 years, hazard ratio (HR) 1.339, 95 % confidence interval (CI) 1.269-1.412), race(black, HR 1.045,95%CI 0.971-1.123; others, HR 0.785, 95%CI 0.735-0.839), pathological grading(Grade III/IV, HR 1.430, 95% CI 1.349-1.517; unknown, HR 1.208, 95 % CI 1.074-1.358),histologic type (mucinous/signet ring cell, HR 1.175, 95% CI 1.113-1.241), SEER stage(Regional, HR 3.379, 95% CI 3.153-3.622; Distant, HR 7.269, 95% CI 6.725-7.857;Unstaged, HR 3.473, 95% CI 2.781-4.361), marital status(widowed, HR 1.290,95 %CI 1.205-1.382; single, HR 1.127,95 %CI 1.048-1.213; divorced/separated, HR1.082, 95% CI 0.994-1.179).

### Subgroup analysis for evaluating the effect of marital status according to SEER stage

We then made further analysis of the effects of marital status on survival in each tumor stage. We observed three interesting findings. First, marital status was an independent prognostic factor in each tumor stage both in univariate and multivariate analysis (*P* < 0.05). Second, patients in the widowed group always had the lowest survival rate when compared with patients in the other groups. Widowed patients had 7.1% reduction in 5-year CSS compared with married patients at Localized stage (77.2% *vs* 70.1%, *P* < 0.001), 9.6% reduction at Regional stage II (38.2% *vs* 28.6%, *P* < 0.001), and 4.7% reduction at Distant stage (13.3% *vs* 8.6%, *P* < 0.001). Third, the difference between the divorced/separated and married group was not apparent in all stage. (Table 3, Figure 1b-1d).

**Table 3:** Univariate and multivariate analysis of marital status on gastric cancer cause specific survival based on different cancer stage

		Univariate analysis		Multivariate analysis	
Variable	5-year CCS	Log rank χ^2^ test	*P*	HR(95%CI)	*P*
**SEER Stage**					
**Localized**					
**Marital status**		25.356	<0.001		
Married	77.2%			Reference	
Widowed	70.1%			1.411(1.202-1.655)	<0.001
Never married	74.6%			1.185(0.974-1.442)	0.090
Divorced/Separated	75.0%			1.136(0.915-1.410)	0.247
**Regional**					
**Marital status**		54.197	<0.001		
Married	38.2%			Reference	
Widowed	28.6%			1.267(1.156-1.388)	<0.001
Never married	34.8%			1.181(1.073-1.301)	<0.001
Divorced/separated	34.1%			1.081(0.964-1.213)	0.184
**Distant**					
**Marital status**		20.161	<0.001		
Married	13.3%			Reference	
Widowed	8.6%			1.226(1.065-1.411)	0.005
Never married	15.7%			1.001(0.868-1.155)	0.984
Divorced/separated	11.0%			1.049(0.894-1.231)	0.557

*P*-values refer to comparisons between two groups and were adjusted for primary site, age, race, pathological grading, and tumor histologic type as covariates.

NI: not included in the multivariate survival analysis.

## DISCUSSION

This study is the first to show an independent beneficial effect of marriage on survival in gastric cancer. The beneficial effect of being married persisted even after being adjusted for stage, age, histologic type, and grade in multivariable analyses. Moreover, widowed patients were always most likely to die of gastric cancer than other groups. Specifically, patients in the widowed group had more common site of stomach, more tumors at localized stage, and higher percentage of adenocarcinoma, all of which were validated as protective prognosis factors in survival analysis. Interestingly, delayed diagnosis was considered as another reason for poor prognosis in unmarried patients [6, 14, 15]. However, in our study group, the percentage of patients with gastric in Localized stage was highest in the widowed group with 39.6% compared with 34.4%, 32.4%, and 34.7% in the married, single, and divorced/separated group, respectively. Obviously, this result is paradoxical given the poor survival outcomes in the widowed group.

Our data revealed that unmarried patients had a survival disadvantage that persisted in each SEER stage. The relationship between marital status and survival can be explained hypothetically by psychosocial factors that are independent of tumor characteristics and extent of treatment. Depression has been reported widely existing among cancer patients [16-18]. Depressive disorders affect up to 38% of patients with cancer, worsen over the course of treatment, persist long after cancer therapy has concluded, and often reappear on cancer recurrence [19, 20]. The prevalence of depression was high in stomach cancer patients even after the completion of treatment, especially among those with problems amenable to treatment [21]. It has been proposed that decreased psychosocial support and psychological stress alter immune function and contribute to tumor progression and mortality [22-24]. Levy et al. reported that a perceived lack of social support was associated with lower activity of natural killer cells [25]. Chronic stress may elicit prolonged secretion of cortisol [26], which triggers a counterregulatory response of white blood cells by downregulating their cortisol receptors. This downregulation, in turn, reduces the cells’ capacity to respond to anti-inflammatory signals and allows cytokine-mediated inflammatory processes to flourish [27], which have been validated as poor prognostic factors in gastric cancer [28, 29]. Conversely, cortisol levels seem to be lower in patients with cancer who have adequate support networks, and diurnal cortisol patterns have been linked with natural-killer cell count and survival in patients with cancer [30, 31]. Additionally, depression and quality of life are related to VEGF, which may stimulate endothelial cell migration, proliferation and proteolytic activity [32]. Unrecognized clinical depression is strongly associated with poor adherence to medical treatment [33]. To date, two prospective studies regarding the association between depression and survival in patients with gastric cancer have been reported [34, 35]. Chen et al. found subjects with higher depression scores had an poor survival compared with the subjects with lower scores [34]. Yu et al. followed 300 patients with gastric cancer and found mortality were higher in patients with depression [35]. The loss of social support or the inability to cope with stress in the widowed groups seems very apparent, and may lead to excess mortality [36, 37].

The results of this study must be interpreted in the light of certain limitations. First, the SEER database only provides the marital status at diagnosis. There is potential for misclassification of marital status. We did not take into account changes of marital status that may have occurred during the follow-up period, which may have influenced outcomes. Thus, our findings may underestimate the protective effect marriage has on gastric cancer outcome. Second, SEER database lacks information of education, income status, insurance status, socioeconomic status and quality of marriage, which might confound the explanation of the disparity in survival between marital groups. For example, marital distress has long-term immune consequences and enhances the risk of a variety of health problems [38]. Third, information on therapy options (radical resection or palliative therapy), subsequent therapy, co-morbidities and recurrence is also lacking.

Despite these potential limitations, results of our study confirmed that unmarried patients are at greater risk of cancer-specific mortality. Especially, widowed patients were always at the highest risk of death of cancer than those in other groups. We concluded that much of the benefit enjoyed by married women is derived from intrinsic social support and social networks. The value of this finding is that social support may well be amenable to intervention and may lead to improved outcomes [39]. Health care providers should recognize that the unmarried patients are at particular risk with respect to treatment of, and survival from gastric cancer. These patients may require more counseling and comprehensive case management.

## MATERIALS AND METHODS

### Patient selection in the SEER database

Frequency and survival data were obtained from the SEER Program database using SEER*Stat 8.1.5 software (National Cancer Institute, Bethesda, MD); specifically, the SEER 18 dataset (consisting of 18 registries, covering the years (2004-2012) was used. The current SEER database consists of 18 population-based cancer registries that represent approximately 28% of the population in the US. It uncover sensitive patient information and is widely used for studies of the relationship between marital status and survival outcomes of patients with cancer [6, 7, 9, 11, 40, 41].

Using the SEER-stat software (SEER*Stat 8.1.5), we searched for patients diagnosed between 2004 and 2012 with single primary gastric cancer and a known marital status. Histological types were limited to adenocarcinoma, mucinous adenocarcinoma, and signet ring cell carcinoma. Patients were excluded if age at diagnosis was less than 18 years, had more than one primary cancer but the gastric wasn't the first one, had no surgical resection, had unknown cause of death or unknown survival months.

### Statistical analysis

Data was analyzed based on age, gender, race, histologic grade, tumor location, extent of disease, and treatment (surgical resection or not). Race was divided into white, black, and others. According to the SEER staging system, tumors that remain *in situ* or confined to the organ are regarded as localized. Those that locally invade or metastasize to regional lymph nodes are considered to be regional, whereas those that travel to distant organs are categorized as distant. Within the SEER database, marital status of the patient is recorded at the time of diagnosis. Marital status is coded as married, divorced, widowed, separated, never married, and unmarried or Domestic Partner. Individuals in the separated and divorced group were clustered together as the divorced/separated group, never married, and unmarried or Domestic Partner were grouped as single group in this study.

Patient baseline characteristics were compared with the χ2 test, as appropriate. The rate of death was compared between groups using the Kaplan-Meier method. Multivariable Cox regression models were built for analysis of risk factors for survival outcomes. The primary endpoint of this study was CSS, which was calculated from the date of diagnosis to the date of cancer specific death. Deaths attributed to gastric cancer were treated as events and deaths from other causes were treated as censored observations. All of statistical analyses were performed using the statistical software package SPSS for Windows, version 17 (SPSS Inc, Chicago, IL, USA). Statistical significance was set at two-sided *P* < 0.05.
